# Vitrectomy versus scleral buckle for retinal detachment without posterior vitreous detachment

**DOI:** 10.1038/s41598-024-67318-w

**Published:** 2024-07-25

**Authors:** Ioanna Dimakopoulou, Georgios Mylonas, Johannes Iby, Alexandra Sedova, Marlene Hollaus, Stefan Sacu, Michael Georgopoulos, Ursula Schmidt-Erfurth

**Affiliations:** Department of Ophthalmology and Optometry, Medical University of Vienna, Währinger Gürtel 18-20, 1090 Vienna, Austria

**Keywords:** Retinal detachment, Posterior vitreous detachment, Vitrectomy, Scleral buckling, Diseases, Medical research

## Abstract

To compare the effectiveness and safety of scleral buckling and pars plana vitrectomy in treating retinal detachment without posterior vitreous detachment. A total of 88 eyes of 83 patients with retinal detachment without prior posterior vitreous detachment were investigated retrospectively. Group A comprised patients who underwent scleral buckling (n = 47) and Group B (n = 36) patients who were treated with pars plana vitrectomy. Anatomical success, postoperative visual acuity, and ocular adverse events were evaluated. The primary and final anatomical success rate showed a nonsignificant difference (*p* = 0.465 and *p* = 0.37 respectively). No significant difference was observed in the reoperation rate or development of epiretinal membrane between the groups (*p* = 0.254 and *p* = 0.254 respectively). However, scleral buckling resulted in significantly better visual acuity at the last follow-up (0.12 ± 0.23) compared to pars plana vitrectomy (0.37 ± 0.46, *p* = 0.001). The incidence of cataract progression was also significantly higher in the pars plana vitrectomy group (46%) compared to the scleral buckling group (10%, *p* < 0.001). Scleral buckling and pars plana vitrectomy show similar success rates in treating retinal detachment without vitreous detachment. However, due to less cataract progression and better visual acuity outcomes, scleral buckling is recommended for these cases. Determining vitreous status before surgery is crucial for optimal outcomes.

## Introduction

The incidence of retinal detachment (RD) in Europe is approximately 1 per 10.000 inhabitants per year and exhibits a peak in the sixth and seventh decade of life^1,2^. Additionally, over the past few years, there has been an increase in the incidence of RD, particularly in males who are 50 years of age or older^3^.

Rhegmatogenous retinal detachment is the result of vitreous fluid passing through a retinal break and separating the outer segments of the photoreceptors from the retinal pigment epithelium (RPE)^4^. For an RRD to happen, three criteria need to be fulfilled: vitreous gel liquefaction; an anomalous posterior vitreous detachment, defined as partial detachment of the vitreous from the retina; and a tear in the retina^5^.

A retinal break is considered a full-thickness defect in the neurosensory retina, and it usually develops due to a tear in the retina after posterior vitreous detachment (PVD)^6,7^. Lattice degeneration can also cause other forms of retinal breaks called round or atrophic retinal holes^8^. Other causes of retinal breaks are trauma and inflammation^7^.

Non-PVD RD is a special form of RD in which a retinal tear and detachment are present despite the vitreous appearing to be intact. Non-PVD RD is typically associated with either retinal dialysis or round retinal holes and is more common in young myopic patients, in whom vitreous liquefaction has still not taken place. An overlying pocket of liquefied vitreous can gradually seep through these holes, leading to detachment^9–11^.

The most common surgical treatments for rhegmatogenous retinal detachment are pars plana vitrectomy (PPV) and scleral buckling (SB)^12^. Recent advances have also introduced the combination of PPV and SB, which leverages the benefits of both techniques^13^. Additionally, pneumatic retinopexy is another method used in certain cases, involving the injection of a gas bubble to reattach the retina^14^.

Although many studies compare PPV and SB for the treatment of uncomplicated RD, there is still no consensus on the best approach^12,15–17^. While PPV is generally preferred in pseudophakic patients, SB is the surgical method of choice in young phakic patients^12,18^. However, the status of the vitreous was not specified in most of these studies. Hence, our study aims to compare the surgical outcomes of SB and PPV in eyes with RD without PVD.

## Methods

Data from consecutive patients undergoing either PPV or SB for RD between 2008 and 2021 at the Department of Ophthalmology and Optometry, Medical University of Vienna, Austria, were retrospectively analyzed in this clinical study. The herein study was approved by the institutional review board of the Medical University of Vienna and was conducted according to the tenets of the Declaration of Helsinki. In addition, all surgeries were performed by 6 experienced retinal surgeons.

In our study, we obtained informed written consent from the participants prior to their inclusion in the research. For minors, informed consent was obtained from a parent or legal guardian. In addition, all data collected from participants were fully anonymized before they were accessed.

Patients with a minimum age of 11 years old and a rhegmatogenous or chronic RD, or an RD due to macular schisis, without accompanying vitreous detachment were included. Exclusion criteria comprised detachment following globe perforation.

**Figure 1:**
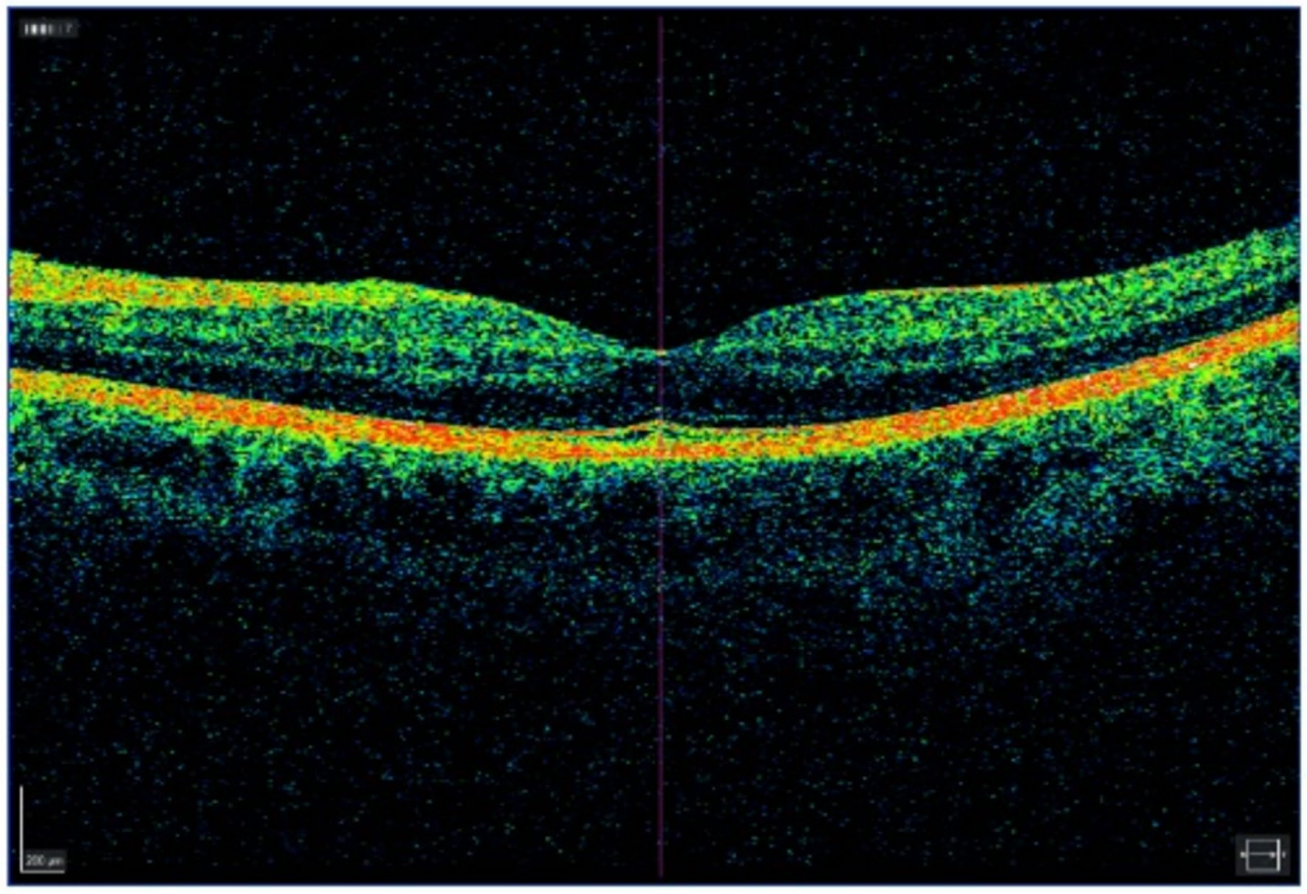
Preoperative OCT image of a patient with non-posterior vitreous detachment rhegmatogenous retinal detachment. The posterior vitreous surface appears to be stilll attached to the fovea.

Data collected during the preoperative control after slit lamp examination were submitted on a Case Report Form (CRF), and optical coherence tomography (OCT) images stored in the hospital’s information management platform were also examined. Additionally, the condition of the vitreous was verified during a slit lamp examination with dilated pupils, specifically checking for indications of a Weiss ring or other signs of posterior vitreous detachment (PVD). Optical coherence tomography (OCT) was also utilized to determine whether the posterior vitreous surface was attached to the fovea (VMA) or solely connected to the optic nerve, indicating a complete PVD (Fig. 1).

Patients were divided into two groups, according to the type of surgery they underwent. Group A included patients who underwent scleral buckling (encircling or segmental) and Group B comprised patients who underwent pars plana vitrectomy.

### Surgical techniques

#### Segmental scleral buckling

The scleral buckling procedure was performed as follows: A circural incision was made in the conjunctiva at the limbus, rectus muscles were lifted with forceps allowing placement of transconjunctival traction 4–0 silk sutures. The sclera was exposed. Under indirect ophthalmoscopy, cryopexy was applied on defects found, after the sclera was marked with a dye on the position of the defect. A scleral explant (radial or circumferential sponge) was fixated with 4–0 mersilene sutures. External drainage was performed in cases where it was considered necessary. The sutures were then tightened and it was ensured that the buckle was well indented. The conjunctiva was then closed with 8–0 vicryl sutures.

#### Encircling scleral buckling

The initial steps were the same as in the segmental buckling procedure. After the placement of the mersilene sutures in all four quadrants of the equator, a encircling silicone band was inserted, with the “lock” positioned superior and nasal. External drainage was performed in cases where it was considered necessary and the band was then further tightened. The correct positioning of the band was verified, as well as the indentation.

#### PPV

Three 23-gauge ports were placed in a typical manner with the infusion placed temporal and inferior. Using the BIOM system, the retinal detachment was observed. A complete circular vitrectomy was then performed and the posterior pole was stabilized using perfluorocarbon liquid (PFCL). The attached vitreous was carefully lifted from the posterior pole, either immediately or after triamcinolone utilization to enhance the visualization of the posterior cortical vitreous. Cryopexy or endolaser coagulation was applied to defects found intraoperatively. The entire peripheral retina was examined with indentation. Fluid-air exchange was performed and PFCL was aspired from the posterior pole. Air was exchanged with either SF6, C2F6 or C3F8 diluted gases. The 23-gauge ports were removed and the sclerotomies were closed with 8–0 vicryl sutures.

Information about patients’ previous/simultaneous RD on the fellow eye as well as type and duration of symptoms were collected. The following RD characteristics were documented: size, expansion, height, grade of PVR, number, type, and size of defects.

Additionally, primary and final success rates, best corrected visual acuity (BCVA) at 1, 3, and 6 months postoperatively and last visit, as well as complications including re-operations, cataract development, PVR, and epiretinal membrane development were compared between the two groups.

The visual acuity was obtained in decimal form and was converted to the logarithm of the minimal angle of resolution (logMAR).

Localized small subretinal fluid (SRF) observed on OCT with no increase during follow-up was not considered a surgical failure. Single-surgery anatomic success (SSAS) was defined as the complete reattachment of the retina after one surgery, not requiring any additional retinal procedures until the end of the follow-up period.

Patients’ data were collected in an Excel database and converted to SPSS for statistical analysis. Continuous variables were presented as mean ± standard deviation (SD). The chi-square test and independent-samples t-test were used as appropriate, with a *p* value of 0.05 considered statistically significant. Additionally, multiple linear regression analysis was performed to further investigate the factors influencing BCVA outcomes. This regression model included variables such as surgical technique (buckling vs. PPV), follow-up duration, age, and preoperative BCVA. Subgroup analysis was also conducted for phakic patients aged 25 to 51 to identify specific effects within this demographic.

## Results

### Participants’ characteristics

Data collected from 1917 patients receiving surgery for retinal detachment between 2008 and 2021 showed that only 88 eyes (4%) were found to be eligible for this study. Table 1 summarizes the demographic data and clinical characteristics of the patients.

**Table 1:** Demographic data and clinical characteristics.

	Total Population	Group A	Group B	*p*-value
No. of eyes	88	49	39	
No. of patients	83	47	36	
Males	46	24	22	
Females	37	23	14	
Sex male to female (m:f)	1	1	2	0.361
Mean age	36 ± 15	31 ± 11	43 ± 16	** < 0.001**
Follow-up after first surgery (months)	17 ± 21	18 ± 20	17 ± 25	0.846
Bilaterality	11	4	7	0.141
Lens status (baseline)				0.02
Phakic	81	49	32	
Pseudophakic	7	0	7	
Refraction				0.354
emmetropia	19	9	10	
myopia	64	38	26	
hypermetropia	2	0	2	

Significant values are in (bold).

The present study included 83 patients, 88 eyes with primary RD, 49 eyes undergoing SB (Group A), and 39 eyes receiving PPV (Group B).

The total cohort comprised 46 male and 37 female patients with a mean age of 37 ± 15 years. The mean follow-up time was 17 ± 21.2 months.

The rate of bilaterality was found to be 13% (11 out of 83 patients) during our follow-up. Out of those 11 patients with bilateral retinal detachment, only 5 (6%) exhibited non-PVD RD in both eyes, which were included in our study. All of these patients were treated with the same technique in both eyes.

64 eyes (73%) of all cases were myopic, with 38 (78%) eyes in Group A and 26 (67%, *p* = 0.354) eyes in Group B exhibiting a refractive error of at least 2 diopters (dpt).

A significant difference in the patients’ mean age (*p* < 0.001), as well as the lens status (p = 0.02), was found between Group A and Group B. The mean age in Group A was 31 ± 11 years, while in Group B it was 43 ± 16 years. Regarding lens status at baseline, all 49 patients (100%) in Group A were phakic, compared to 32 patients (82%) in Group B.

### Retinal detachment characteristics

Table 2 summarizes the RD characteristics.

**Table 2:** Retinal detachment characteristics.

	Total population		Group A		Group B		*p*
Retinal detachment characteristics							
Duration of RD (days)	22 ± 47		16 ± 19		29 ± 69		0.227
No. of fovea-off detachments	33		19		14		0.782
Duration of fovea off (days)	28 ± 69		10 ± 21		52 ± 100		0.042
Quadrants (Mean/SD)							0.557
1	29	33%	18	37%	11	28%	
2	40	45%	23	47%	17	44%	
3	14	16%	6	12%	8	21%	
4	3	3%	1	2%	2	5%	
Height of RD							0.319
Flat	68	77%	41	84%	27	69%	
High	15	17%	6	12%	9	23%	
PVR							0.770
None	37	42%	21	43%	16	41%	
PVR-A	21	24%	13	27%	8	21%	
PVR-B	20	23%	11	22%	9	23%	
PVR-C	7	8%	3	6%	4	10%	
Retinal defects							
Number of defects (No., Stdev)	2 ± 1		2 ± 1		2 ± 2		0.873
Size of defects median (in clock hours)	0.5		0.5		0.5		
Type of defect							0.686
Round hole(s)	64	73%	36	73%	28	72%	
Horseshoe tear	8	9%	4	8%	4	10%	
Giant tear	1	1%	1	2%	0	0%	
Break in the ora serrata	2	2%	1	2%	1	3%	
Retinoschisis	4.0	5%	1.0	2%	3.0	8%	
No defect found	4.0	5%	2.0	4%	2.0	5%	
Undefined	5.0	6%	4.0	8%	1.0	3%	
Location of defect							0.311
Superotemporal	31.0	35%	13.0	27%	18.0	46%	
Inferotemporal	34.0	39%	24.0	49%	10.0	26%	
Inferonasal	5	6%	3	6%	2	5%	
Superonasal	8	9%	4	8%	4	10%	
Everywhere	5	6%	3	6%	2	5%	

The average duration of RD, from the onset of symptoms till the patient’s first medical consultation, was 16 ± 19 days for Group A and 29 ± 69 days for Group B (p = 0.227). Fovea-off detachments did not differ in number between the groups (Group A = 19, Group B = 14, p = 0.578). However, they did differ in duration (average duration of fovea-off detachment in days: Group A = 10 ± 21, Group B = 52 ± 100, *p* = 0.042). The medians of time to the first consultation for this group (2 days for Group A and 6 days for Group B) suggest influence of outliers and the statistical difference in duration was not significant according to the Mann–Whitney U test (*p* = 0.096).

On average, patients in Group A demonstrated 2 ± 1 defects and patients in Group B had 2 ± 2 (*p* = 0.873). The size of the defects did not differ between the groups (Group A Median = 0.50 clock hours, Group B Median = 0.50 clock hours).

The most commonly found defect types were round holes, either in groups or individual (Group A = 36 (73%), Group B = 28 (72%)), followed by horseshoe tears (Group A = 4 (8%), Group B = 4 (8%)). Two breaks in the ora serrata were found in total, one in each group, as well as a giant tear in Group A. Four patients exhibited a retinoschisis, 1 in Group A and 3 in Group B. In 5 cases in total the defect was not defined and in 4 cases no defect was found.

Supertemporal retinal defects were found in 31 (35%) eyes (13 in Group A and in 18 in Group B respectively), inferotemporal defects were found in 34 (39%) eyes (24 in Group A and 10 in Group B respectively), superonasal defects were found in 8 (9%) eyes (4 in Group A and 4 in Group B respectively), and inferonasal defects were found in 5 (6%) eyes (3 in Group A and 2 in Group B respectively). In 5 (6%) eyes (3 in Group A and 2 in Group B respectively) defects were located in all quadrants of the retina.

In Group A, 34 patients (69%) received a segmental buckle, while 15 patients (31%) received a circumferential buckle. In Group B, a total of 39 patients underwent pars plana vitrectomy (PPV). Among these, 35 eyes (90%) received PPV with gas injection, 4 eyes (10%) received PPV with silicone oil. More specifically, 12 patients received SF6 gas, 22 patients received C3F8 gas and 1 patient received C2F6 gas.

### Best-corrected visual acuity

Table 3 shows the progression of BVCA from baseline till the last follow-up.

**Table 3:** Progression of BVCA from baseline till the last follow-up.

Month	Group A	Group B	*p* value
Baseline	0.42 ± 0.57	0.58 ± 0.65	0.235
Month 1	0.32 ± 0.37	0.44 ± 0.59	0.292
Month 3	0.21 ± 0.30	0.43 ± 0.48	**0.023**
Month 6	0.18 ± 0.24	0.39 ± 0.55	0.068
Last follow up	0.12 ± 0.23	0.37 ± 0.46	**0.001**

Significant values are in (bold).

The preoperative BCVA was 0.42 ± 0.57 logMAR in Group A and 0.58 ± 0.65 in Group B (*p* = 0.235).

There was no significant difference in BVCA between the two groups at 1 and 6 months of follow-up (BCVA month 1: Group A: 0.32 ± 0.37 logMAR, Group B: 0.44 ± 0.59 logMAR, *p* = 0.292; BCVA month 6: Group A: 0.18 ± 0.24 logMAR, Group B: 0.39 ± 0.55 logMAR, p = 0.068). However, BCVA was significantly better in Group A at month 3 (BCVA month 3: Group A: 0.21 ± 0.30 logMAR, Group B: 0.43 ± 0.48 logMAR, *p* = 0.23) and at the last follow-up (Group A: 0.12 ± 0.23 logMAR, Group B: 0.37 ± 0.46 logMAR; *p* = 0.001).

A linear regression analysis was conducted to assess the impact of surgical technique, age and preoperative BCVA on postoperative BCVA outcomes. The regression model revealed that preoperative BCVA was a highly significant predictor of postoperative BCVA (0.377 [0.270; 0.485], *p* < 0.001) indicating that higher preoperative BCVA values were strongly associated with higher postoperative BCVA values. However, the type of surgical procedure (buckling vs. PPV) did not significantly affect postoperative BCVA (0.049 [− 0.091; 0.188], *p* = 0.490) and neither did age (0.001 [− 0.004; 0.005], *p* = 0.798).

### Complications

All complications observed are shown in Table 4.

**Table 4:** Complications.

Complications	Total		Group A		Group B		*p*-value
None	40	45%	27	55%	13	33%	
Cataract development	23	26%	5	10%	18	46%	** < 0.001**
ERM	6	7%	2	4%	4	10%	0.254
Translocation of scleral buckle	1	1%	1	2%	–		
Reoperation	15	17%	6	12%	9	23%	0.288

Significant values are in (bold).

Out of 81 phakic eyes at study entry, 23 (26%) underwent cataract surgery during the postoperative course. Cataract development and progression were observed in 5 (10%) eyes in Group A and 18 (46%) in Group B (*p* =  < 0.001). Epiretinal membranes (ERM) were observed in 2 eyes in Group A (4%) and 4 eyes in Group B (10%) (*p* = 0.254). In addition, a translocation of the scleral buckle was observed in one patient (2%) in Group A.

After running a regression analysis, we found that the type of operation (buckling vs. PPV) was a significant predictor of complications. The model indicated that patients who underwent PPV had significantly higher odds of experiencing cataract progression compared to those who underwent buckling (OR = 8.170, *p* < 0.001).However, the type of operation was not found to be a significant predictor when examining for the development of ERM.

### Anatomical outcomes

Primary anatomical success was achieved in 44 out of 49 (90%) eyes in Group A and 33 out of 39 (85%, *p* = 0.465) eyes in Group B.

PVR was responsible for 7 of 16 redetachments in total, including 2 out of 7 redetachments in Group A and 5 out of 9 redetachments in Group B (*p* = 0.377). Unrecognized or new retinal breaks were causative for 5 out of 8 redetachments in Group A and 3 out of 9 in Group B. The cause could not be identified in the rest of the cases.

Within Group A, 7 (14%) patients underwent a reoperation, 6 of which PPV combined with gas injection, and one patient received anterior chamber puncture with intravitreal SF6 gas injection. In contrast, among the 9 (23%) patients in Group B who required a reoperation, five individuals received scleral buckling, three underwent PPV with gas injection, while one patient underwent PPV with silicon oil injection.

Final anatomical success was achieved in 48 of 49 eyes (97.96%) in Group A and in 39 of 39 eyes (100%) in Group B. (*p* = 0.37).

### Subgroup analysis

In this study, we conducted a subgroup analysis focusing on patients with intact lenses (phakic patients) within a specific age range (25–51 years old). We compared the outcomes between two groups, labeled as Group C for patients that received buckling and Group D for patients that received pars plana vitrectomy.

A total of 54 eyes could be added in this subgroup, 31 eyes in Group C and 23 in Group D. The mean age of patients in both groups did not show any statistically significant difference (*p* = 0.728).

While preoperative BCVA was not significantly different between the two subgroups (Group C: 0.25 ± 0.42 logMAR, Group D: 0.39 ± 0.52 logMAR, *p* = 0.25), postoperative BCVA exhibited a statistically significant difference even between the subgroups. Group C achieved a mean BCVA of 0.04 ± 0.08 logMAR; while Group D recorded a mean BCVA of 0.31 ± 0.48 logMAR (*p* < 0.001). A linear regression analysis was also conducted on the subgroup of phakic patients aged 25–51 years, to assess the impact of surgical technique on postoperative BCVA outcomes. The regression model revealed that preoperative BCVA was a highly significant predictor of postoperative BCVA (*p* < 0.001). The type of surgical procedure was also a significant predictor (*p* = 0.007), with buckling associated with significantly better postoperative BCVA outcomes compared to PPV.

Regarding the reoperation rate, we found no significant distinction between the two groups, as Group A had 6 reoperations, and Group B had 4 (*p* = 0.254).

Moreover, our subgroup analysis indicated a higher incidence of cataract progression in Group D, the vitrectomy group. Specifically, 10 patients from Group D required cataract operations, while four patients from Group C underwent a cataract operation (*p* < 0.001).

## Discussion

The aim of this retrospective study was to focus on a special type of retinal detachment, occurring in the absence of PVD, as well as to compare two surgical methods retrospectively for the treatment of these patients.

Data from 1917 patients undergoing retinal detachment surgery between 2008 and 2021 revealed that only 88 eyes (4%) were eligible for this study, highlighting the rarity of non-PVD RD. As noted by Mitry et al. over 85% of RRD cases involve PVD and tractional tears^10^.

To our knowledge, there have not been any previous studies comparing these two surgical techniques for the treatment of RD, after defining the status of the vitreous.

It was indicated that both scleral buckling and PPV can achieve similar single surgery success as well as final anatomical success rates in those patients. The reoperation rate was also found to be non-different between the two groups. Nevertheless, BCVA at the last follow-up was found to be significantly better in Group A than in Group B (*p* = 0.001). In addition, the cataract progression rate (*p* < 0.001) was significantly higher in Group B.

In the Scottish Retinal Detachment Study, PVD-absent RD showed a slight male predominance (60% vs. 40% 5.6, *p* = 0.017), while studies regarding RD secondary to round retinal holes prove a female predominance^9,10,19^. We did not find a gender predominance documented in non-PVD RD, with 46 out of 83 (55%) patients being male and 37 out of 83 patients (45%) being female.

The bilaterality rate of non-PVD RD in the general RD population is around 10%, while non-PVD RD associated with atrophic holes exhibits a much higher rate, ranging from 30 to 45% in two studies, conducted by Gonzales et al. and Ung et al. respectively^9,20–23^. In our study, 13% of patients suffered from RD in both eyes, which is more in line with the general population.

In our total cohort, we observed a high rate of myopia (64 out of 88 eyes were myopic). Myopia incidence in RD patients in various epidemiological studies and different populations ranges from 46.9 to 66.5%^2,21,22,24^. This is a finding supporting previous observations that RD cases without PVD tend to be more myopic than PVD-related RDs^9^.

The significant difference in the patients’ mean age (*p* < 0.001), as well as the lens status (*p* = 0.02) can most likely be explained due to the fact that PPV is often the method of choice in older patients, who have already undergone cataract surgery. However, it is important to note that even when considering only patients with intact lenses (phakic patients) and adjusting for age our subgroup analysis yielded similar outcomes.

The most common type of defect found at baseline was round holes, individual or in groups (73% of eyes). Given that PVD is not present, round atrophic holes are the most common etiopathological factor responsible for retinal detachment in these patients^9,25^. A different pathogenic mechanism is probably responsible for the RD in the rest of the patients, who exhibited horseshoe tears (9%), a giant retinal tear (1%), and two ora serrata tears (2%).

BCVA was significantly lower in Group B at the last follow-up. While the regression analysis on the overall cohort showed no significant effect of surgical technique (buckling vs. PPV) on BCVA (*p* = 0.658), the subgroup analysis revealed that PPV was associated with poorer BCVA outcomes compared to buckling (*p* = 0.007). This suggests that the impact of surgical techniques may be more pronounced in younger, phakic patients.

On the other hand, a lower BCVA in Group B could also be attributed to cataract progression, which tends to be faster after PPV. However, all of the patients were already operated for cataract, by the time a final BCVA was acquired^17^. Furthermore, SB has been shown to achieve significantly better final BCVA in other studies, as well as better postoperative functionality of the preoperatively attached areas compared to PPV^12,16,26,27^.

Our results showed postoperative cataract progression in 46% of the patients in Group B and 10% in Group A (< 0.001). All of these patients underwent cataract surgery postoperatively. Heimann et al. reported a cataract surgery rate after 1-year follow-up of 58% in the PPV group and 20.6% in the SB group, which was also significant and corresponds well with our results^16^.

Further, the ERM development in our study did not differ in a statistically significant trend between the two groups (*p* = 0.254). In a recent study, ERM development during follow-up was found to be 28% in the SB Group and 34%in the PPV Group, respectively, and again was not significant between the two groups (*p* = 0.536)^28,29^.

In our study population, PVR development was the cause of redetachment in 7 out of 16 (44%) eyes. More specifically, 2 out of 7 (29%) redetachments in Group A and 5 out of 9 (56%) redetachments in Group B were due to PVR (*p* = 0.377). Rates of 16% to 66% have been described for proliferative vitreoretinopathy-related surgical failure after PPV and 39% for scleral buckling^30–32^.

Nonetheless, there are some limitations to our study. Due to its retrospective nature, patients could not be randomized and there were discrepancies in the baseline characteristics. Group A patients were significantly younger than Group B (*p* < 0.001), which is easily explained by the fact that SB is the preferred method of surgery in younger patients. Furthermore, patients in Group B exhibited a greater rate of pseudophakia (*p* = 0.02). These characteristics are related to a poor prognosis. To mitigate the impact of these differences, we conducted a subgroup analysis to adjust for these baseline disparities. The subgroup analysis findings supported the overall results, reinforcing our conclusions. Other weaknesses include a short follow-up period in some patients and a small number of patients.

In conclusion, both SB and PPV surgery were found to yield similar initial and final anatomical success rates in patients without PVD. Cataract progression was faster in patients who were treated with PPV, and the final BCVA was found to be better in the SB group.

Our suggestion is that the status of the vitreous should always be determined in patients with retinal detachment, and non-PVD RD should be initially treated with SB. PPV should be considered instead in case of revision surgery. A prospective, multicenter, randomized study is warranted for obtaining a more definite conclusion on the role of PPV in non-PVD RD.

## Data Availability

The datasets generated and/or analyzed during the current study are not publicly available due to privacy or confidentiality concerns regarding the sensitive nature of the data. However, they are available from the corresponding author upon reasonable request to ensure responsible handling and protection of sensitive information.
